# Supplementary material for: Effects of population mobility on the COVID-19 spread in Brazil
Source: PLoS One. 2021 Dec 7;16(12):e0260610. doi: 10.1371/journal.pone.0260610 (PMC8651143; doi:10.1371/journal.pone.0260610)
Supplement: Attachment — Submitted filename: report.pdf [file pone.0260610.s001.pdf]

Summary

The manuscript investigates the SARS-CoV-2 spread and its relationship with the policies adopted in Brazil using mobility data, aiming to show how this data can be used to understand the virus propagation. A SENUR compartmental model is used to accommodate such data with parameters to study hypothetical scenarios of mobility restrictions. The authors used aggregated (Waze) and fine-grained (DETRAN-CE) data.

Although I think this data can be useful, I am not convinced that the authors used it in a way to allow to distinguish the main results from a more basic model, without mobility data. As many things were not clear, I suggest a heavy revision of the manuscript, according to the points I make below, together with the fact that I found that the ideas were put in a very confusing way. An even more systematic revision should be done on the figures, which do not follow a style pattern, either without labels or using different names for the same metrics.

One of the PLOS ONE's criteria for publication is that *"Experiments, statistics, and other analyses are performed to a high technical standard and are described in sufficient detail."* I do not see that in the present version of the manuscript. No sensibility analysis was performed to show the present correlations between the parameters defined for the model, which does not allow drawing the conclusions stated by the authors.

Finally, my opinion is that the manuscript should be rewritten with a better organization and statistical analysis to support the conclusions.

General comments

In the abstract, it is stated that "This work is the first to shed light on the pandemic situation on the Brazilian territory using both aggregated (. . .) and fine-grained (. . .) mobility data (. . .)": It is not true. Here I cite some works that investigated this issue in Brazil using these kinds of data:

- "Assessing the potential impact of COVID-19 in Brazil: mobility, morbidity and the burden on the health care system." medRxiv 2020.03.19.20039131 (2020)
- "Evolution and epidemic spread of SARS-CoV-2 in Brazil." Science 369.6508 (2020): 1255-1260.
- "Modeling future spread of infections via mobile geolocation data and population dynamics. An application to COVID-19 in Brazil." PLOS ONE 15.7 (2020): e0235732.
- "Outbreak diversity in epidemic waves propagating through distinct geographical scales." Physical Review Research 2(4) (2020): 043306.
- "Spatiotemporal pattern of COVID-19 spread in Brazil." Science 372.6544 (2021): 821-826.

None of these works are on the reference list.

The mobility restrictions seem to be a change in the infection rate, as a "social distancing" measure or other NPIs. If the authors had mobility data as a function of time (that was not clear to me), such as the data available in

- "Heterogeneous impact of a lockdown on inter-municipality mobility." Physical Review Research 3.1 (2021): 013032.

- "COVID-19 lockdown induces disease-mitigating structural changes in mobility networks." Proceedings of the National Academy of Sciences 117.52 (2020): 32883-32890.

it would be interesting. However, the model is based on a parameter q_t that is calibrated and no sensitivity or calibration analysis was presented to show the possible correlation between different parameters. I am not convinced that the mobility data used were important to draw conclusions.

No discussion was made about each city's results, of how the measures adopted there were efficient or not to mitigate the spread. There is only a discussion about how the numbers would change if different strategies were adopted.

Figures: Each figure uses a different style and notation. Not even the legends of the figures were adapted to fit the space, and in some cases the legend hides the curves or points.

Specific points:

- Abstract) What the authors mean by "data from public sources"? The data is not available publicly and this phrase sounds like it is.
- p.1) "The negative effects of the COVID-19 pandemic in Brazil may be related to the lack of knowledge about the disease and virus characteristics, such as its lethality and high transmissibility": I do not agree with the "lack of knowledge", it may be something else.
- p.2) It is important to distinguish two types of mobility: the flow of people between different areas (such as neighborhoods) and inside each of these areas. At the beginning of the epidemics, interventions of mobility between countries, or even municipalities are important to mitigate the spread from one place to the other by avoiding the mixing of people. Now, however, with cases confirmed in all municipalities and a high number of new cases and deaths every day, it is more important to use a social distancing approach and others NPIs, such as masks to reduce the level of contagion. The mobility can drive the spatio-temporal pattern, but other factors are more important to the local spread. In the way the mobility data was introduced in the model, it seems to be only related to the local spread.
- p.3) Lines 86-88: "shows" -> "show", "we concludes" -> "we conclude", "and discusses" -> "and discuss".
- p.3-4, and in the rest of the paper): Please choose a date format and keep that throughout the text. On page 3, the MM/DD/YYYY is used, then in line 135 the format MM/DD/YYYY is used together with DD/MM/YYYY (probably a typo). Later, in Fig 1c, MM/DD/YYYY is used, but in Figs 5(a-e), 6(a-f) and 10 the YYYY-MM-DD format is used.
- p.4) Is the quotation in lines 118-120 really necessary for the paper?
- p.5) The "Coronavirus Panel data" contains the number of cases and deaths by confirmation or report date, not the notification date as in "Opendata SUS". The first is affected by delays related to inserting the record in the system, the exam collection date, the exam result date, and finally the reporting from the municipality to the state's health department. So, the methodology used by Abbott et al is not enough, in this case, to "rewind" the data by using only the delays from symptoms onset to the notification. It is clear to me when I see Fig. 1(c) and 1(d): the peak around September 2020 appears as a sudden drop in Fig. 1(d). The peak is related to delays in confirming the cases, not in notifying the cases in the system (when the patient seeks medical attention), as the authors correctly stated in lines

178-179. However, I am not convinced that an adequate methodology was used in this case. Please see other methodologies to correct reporting delays, such as

  – "A modelling approach for correcting reporting delays in disease surveillance data." Statistics in Medicine 38.22 (2019): 4363-4377.

- Fig 1: there are no labels in Figs. 1(a,b). What do the y-axis and x-axis mean? In Fig. 1(b), what does the dashed line mean? Why (a) and (b) are in different plots, if (a) is the empirical distribution of the delays and (b) the estimated one? Should not the bars be the empirical distribution and the curve the estimated one? Please clarify.
- p.5, 6, and so on) Another problem with the notation appears in the manuscript: I_{N_i} and I_{U_i} are used in p. 5, but in Eqs. (1-5) they appear as I_{C_i} and I_{S_i}, and I_{C_i} in line 266 (p. 8). Please clarify.
- p.7) μ_i and μ are not defined.
- p.7) "Alternatively, if this information is associated as a function of smaller regions, we can make inferences with finer granularity." What does that mean?
- p.7) Notation problem: here "R_t" is used, but in page 13 it appears as $R(t)$ (and caption of Fig. 7)
- p.7) The value of q_t is calibrated in $W_{ij}(t) = q_t C_{ij}$ to find the values of q_t that better represent the situation in a given time window, correct? The problem is that as μ_i is a free parameter (and not defined), anything can "fit" the real data. Also, it is not clear if C_{ij} is constant in time.
- p.7) The force of infection is $\sum_j \left[W_{ij}(t)\left(\frac{I_{N_i}(t)+I_{U_i}(t)}{N_j}\right)\right]$, meaning that infected individuals from i interact with N_j individuals in j. What is the explanation for that choice? See Sec. 7.2 of

  – Keeling, Matt J., and Pejman Rohani. Modeling infectious diseases in humans and animals. Princeton university press, 2011.

- p.7) In Eqs. (1-2) what is the meaning of the factor $I_{C_i}(t)/N_i$? Has this not already been counted in the infection force? Also, in Eqs. (1-2) it shows $\lambda_{i,t}$, and later as $\lambda_i(t)$.
- p.8) There is no information on the number of cases for each region, correct? I mean, the number of cases is only available at the city level, not by the regions.
- p. 9, Table 1) What are the values of q_t for each case in Table 1? Maybe the median or mean value can be put in the table. The caption text does not describe anything and should be changed. Also, from where were these dates extracted for each city?
- p.9, line 298) There is a typo: "q_1 e q_2" -> "q_1 and q_2".
- p.10, Fig. 4) The caption says that q_1 and q_2 are being shown, but in Fig. 4(b) we have q_3. Please clarify.
- p.10) Were the results compared to a null model discarding the mobility data? I think it is important to state that the mobility data is necessary for the calibration. A calibration or sensitivity analysis is necessary to draw any statistical conclusion. My feeling is that the mobility is not playing a role here: a SEIR model with a time-dependent rate (related to the q_t parameter in this case, and the dates in Table 1), without mobility data whatsoever, can be enough. See

- "A SEIR-like model with a time-dependent contagion factor describes the dynamics of the Covid-19 pandemic." medRxiv 2020.08.06.20169557 (2020)

- p.10, Fig. 5 and simulation results) The confidence interval is very weird. How do the authors explain these piece-wise confidence intervals? Were the data calibrated for each time window with a constant q_t, but a different set of other parameters? If so, that does not seem to be correct, since these time windows are not independent. See Fig. 5(c), for example. Why not plotting also the median or average value of the simulations, instead of only the weird confidence intervals?
- p.11, Fig. 6) The y-axis label seems to be wrong. For q_0, the value is close to 1. Is it the ratio between the number of infected cases reported using this strategy, compared to the calibrated one, or the %? In the caption, it says that it is the ratio, but uses "%" in the label. Please clarify.
- p.11 Fig. 6b) If it is the ratio, why are the values different of 1 when using q_0? What set of parameters was used in this case? Is it a fixed one, since no confidence interval is present?
- p.11 Fig. 6) Why some figures have q_0 and q_2, and others only q_0 in the legends? Please clarify.
- p.11) What the authors mean by "although it is likely to happen" in line 352? What about the new waves of infection with the new variants and mutations?
- p.11) "HDI" is not defined here, only on the next page.
- p.12 and Fig. 7) It was not clear to me if the number of infected people in each region of Fortaleza was obtained from the simulations or if they are real data. If so, what is the source of this data? If it is official reporting data, it does not seems to be related to any mobility data mentioned in the main text.
- p.13) The analysis of the correlation between DETRAN-CE and Google Mobility data is very interesting, especially the lag between $R(t)$ and the time series.
- p.14) I understand that "using a regional approach, we can stratify the information for each region individually". In the caption of Fig. 11, the authors state the number of infected cases was estimated by their model. Without comparison to official reporting data aggregated by region rather than the city as a whole, we cannot conclude anything. If the mobility data is used in a model, it must be compared to official data, or with a null model to show that this was really necessary. Also, again I stress the need for sensitivity analysis of the calibrated parameters.
- p. 14, Fig. 11) Why is Rt so large close to 04/06? No discussion was made about this fact.
- p. 15) My feeling is that similar conclusions for all municipalities investigated could be obtained without using the mobility data. Instead of using W_{ij}, the authors could use, for example, the demographic density to estimate the number of contacts of each region, multiplied by q_t to infer the social distancing and others NPIs.
- p. 15, Fig. 12) Again, the caption says that it is the ratio, but the plot shows "# (Number) of". By the way, I suggest replacing all the "# of" with "Number of".
