# Supplementary material for: Effects of population mobility on the COVID-19 spread in Brazil
Source: PLoS One. 2021 Dec 7;16(12):e0260610. doi: 10.1371/journal.pone.0260610 (PMC8651143; doi:10.1371/journal.pone.0260610)
Supplement: Attachment — Submitted filename: response.pdf [file pone.0260610.s002.pdf]

Response Letter of article PONE-D-21-13982: Effects of population mobility on the COVID-19 spread in Brazil

Eduarda T. C. Chagas, Pedro H. Barros, Isadora Cardoso-Pereira, Igor V. Ponte,
Pablo Ximenes, Flávio Figueiredo, Fabrício Murai, Ana Paula Couto da Silva,
Jussara M. Almeida, Antonio A. F. Loureiro, Heitor S. Ramos

I. Academic Editor

Comment #1

Thank you for submitting your manuscript to PLOS ONE. After careful consideration, we feel that it has merit but does not fully meet PLOS ONE's publication criteria as it currently stands. Therefore, we invite you to submit a revised version of the manuscript that addresses all the points raised in the two excellent reviews.

Thanks for handling this manuscript. We have prepared a revised version taking into account all the comments and suggestions made by the reviewers. We found all the reviews constructive, and we would like to thank the reviewers for helping us make a better contribution.

This response letter addresses all the comments in red, followed by our responses, and, whenever necessary, the changes made (in black). We also include the `diff` article between the prior and current versions, where deletions are in red, and additions are in blue.

II. Reviewer #1

Comment #1

In general, simply put, the plots are barely readable. First, the quality of the images is very poor: even when downloaded the figures appear heavily pixelated with very low resolution. I suggest the authors to use vector graphics systematically.

Secondly, I strongly suggest the authors to arrange multiple plots regarding the same quantity

in large single panels, instead of providing a 10+ pages with one plot for each single page. For instance, the 16 plots for the Rt on different regions could be easily arranged in a single panel with 16 plots.

Also, the figures appear in order 10-11-12-1-2-3-..., which is even more confusing.

Comment #1.1

Fig 1.a: There are no labels in the axes. Also, it is not specified what does the dotted vertical line represent, neither in the plot nor in the text. Mean value? Median? Please, specify.

Fig 1.b: Same as Fig 1a.

Fig 1.c: No need to use a Y scale this large.

Change #1.1

Thanks for the comments. For the sake of readability, we made the following modifications:

- Placed all plots in a single panel,
- Added the captions on x and y axes of Fig 1.a) and 1.b),
- Changed the caption and the text to clarify vertical lines meaning (the mean delay value in each analyzed distribution).

Regarding the scale of plot 1.d, we opt to keep both plots (1.c and 1.d) with exactly same scale to make them directly comparable.

New versions of Fig 1 and caption in Section **Methodology** on Page 5 :

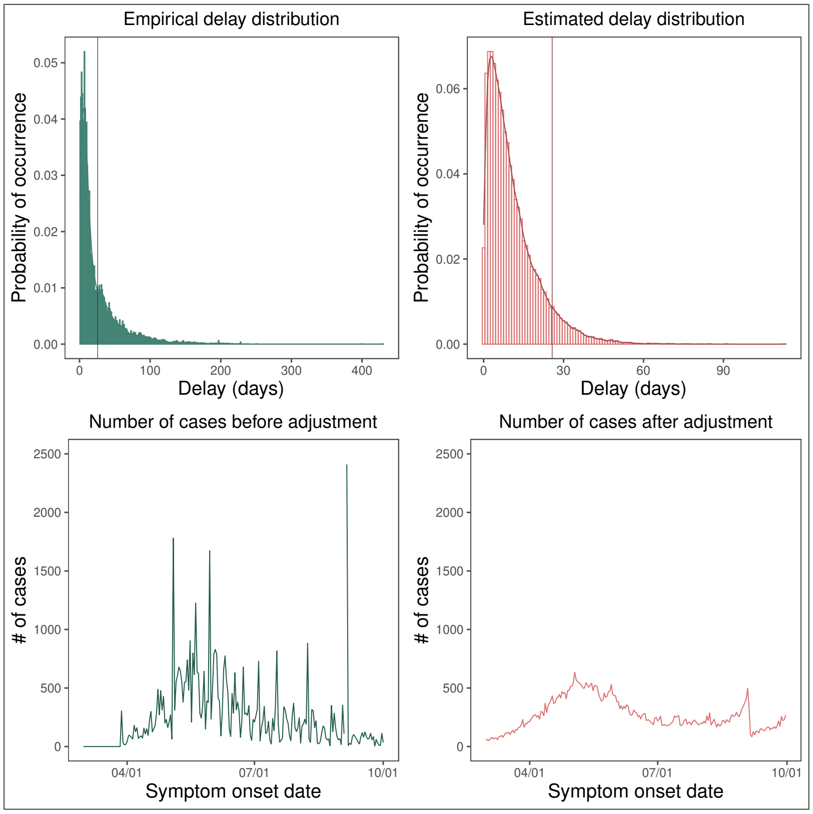

Fig. 1. The distributions of delay and number of cases estimated from the data notified by Opendata SUS platform and the Coronavirus Panel before and after adjustments of the lag between symptom onset and official notification in Fortaleza/CE (Brazil). From right to left, we have: empirical delay distribution, estimated delay distribution, cases distribution before adjustment, cases distribution after adjustment. Vertical lines on first and second plots represent the mean of the delay.

Comment #1.2

Fig 2: Compartments I_{N_i} and I_{V_i} do not appear in the equations in the text. To this matter see also point 4.

Change #1.2

Thanks for pointing this out, we fixed the epidemiological model notation throughout the paper.

Comment #1.3

Fig 5 (all): The legend overlaps the points. Confidence intervals (when they appear), look wrong. The points always sit at the extreme values of the confidence intervals, which does not look correct. My best guess is that one side of the interval is missing, please check the correctness of those confidence intervals.

Change #1.3

We corrected legend positions to avoid overlaps with points in Fig 5 in Section **Case study I: Coarse-grained analysis** on Page 11.

Comment #1.4

Fig 6 (all): Why are the curves discontinuous? Also, the legend in each plot seem to be wrong, for instance in Fig 6a q2 refers to both the red and the green curve. This ambiguity is present in all plots of Fig 6 and gets even worse, for instance in Fig 6c all curves are labeled as q0. Please, clarify or correct.

Change #1.4

Figures show the ratio between the output of our model when considering the real scenario and the result obtained after considering the following two cases:

Scenario I. We consider that the government did not apply any measure to restrict mobility and individuals maintained the same behavior as in the pre-pandemic period. Thus, we are considering here that although the virus was spreading, individuals behavior remained similar to the pre-pandemic period.

Scenario II. The government enforced the closure of the trade soon after the first registered case. Thus, right after period q_0, we apply the mobility corresponding to the period associated with the closing of trade in the city analyzed (q_2 for Fortaleza and Rio, q_4 for São Paulo), following Table 1 dates.

To avoid misinterpretation, we changed the presentation of those plots removing the lines and presenting only the dots. Each dot depicts the one-day prediction of the ratio described above.

We improved the plots quality present in Fig 6 Section **Case study I: Coarse-grained analysis** on Page 11: we re-positioned the legends, changed the ordinate axis label to make it clear that we are working with the ratio between different analyzed scenarios and organized all the figures

in the same panel. Moreover, we removed the lines and presented only the dots.

Therefore, for each period used in scenarios I and II, the average value associated with the parameters estimated in each scenario was used, thus justifying the absence of confidence intervals.

Comment #1.5

Fig 9.a: Missing label on y-axis.

Change #1.5

Thank you for the comment. We added captions on the y axes of Fig 9.a) indicating that such data correspond to the percentage change in the mobility index captured by Google (Section **Case study II: Fine-grained analysis**, Page 14):

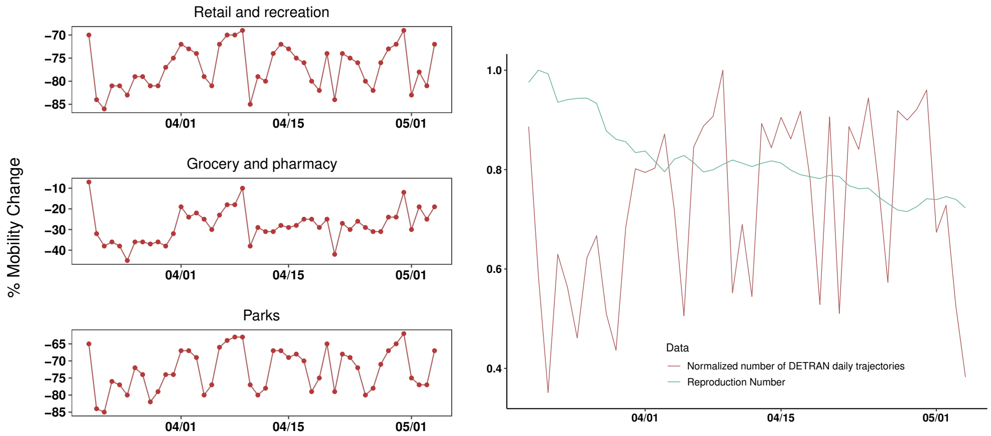

Fig. 9. **Aggregate flow of vehicles and the reproduction number between the dates 03/20/2020 and 05/04/2020.** Plots on the left show mobility indexes extracted from the Google report, and plots on the right depict the $R(t)$ estimated from DETRAN-CE's data. We can see similarities between trends in DETRAN-CE's data and the mobility indexes extracted from the Google report. The highest cross-correlation results are in places labeled as retail and recreation, grocery and pharmacy, and parks.

Comment #1.6

Fig 9.b: What are the unit of the DETRAN line? What does it represent?

Change #1.6

It represents the normalized number of daily trajectories present in the DETRAN/CE's trace. All values were divided by the maximum number of daily trajectories found in the trace, so that all points are in $[0, 1]$. We chose this representation so that we could plot the $R(t)$ values alongside them.

For better understanding the plot in **Section Case study II: Fine-grained analysis** on Page 14, we modified the caption referring to each sequence present in Fig 9.b.

Comment #1.7

Fig 9.b: How (DETRAN data) is computed?

Change #1.7

We consider that if a measurement between two cameras is in the interval $[t_i, t_s]$, the vehicle stopped at a location between the two Automatic License Plate Readers (ALPRs) of DETRAN-CE. We manually investigate these thresholds and we found t_i = 1 hour and t_s = 10 hours.

To facilitate the reader's understanding, we changed the order of the text, adding it as the second paragraph of Section "**Case study II: Fine-grained analysis**". Thus, the reader will already be familiar with the trajectories construction when we analyze these data in detail. Note that t_i and t_s is the difference between passing a vehicle on Detran's radar.

We modified the following paragraph (Section **Case study II: Fine-grained analysis**, Page 12):

> "Our dataset comprises annotations informing when a given vehicle passed by the DETRAN-CE's ALPR sensing location. Thus, for the construction of trajectories, we use the checkpoints of the DETRAN-CE's ALPR as follows. We defined the upper limit (t_s) when a vehicle passes through a DETRAN-CE's ALPR, but no other DETRAN-CE's ALPR identifies it for an extended period. It probably happened due the vehicle used an alternative route that does not have any DETRAN-CE's ALPR from our dataset. Similarly, the lower limit (t_i) is when a vehicle passes two or more DETRAN-CE's ALPR in a short period. This behavior is because the vehicle did not stop anywhere between the DETRAN-CE's ALPRs. Hence, we constructed a trajectory when the measurement between two DETRAN-CE's ALPRs is in the interval $[t_i, t_s]$, indicating that the vehicle stopped at a location between them. We manually investigated the threshold values, and

for this work, we use t_i = 1 hour and t_s = 10 hours."

Comment #1.8

Fig 9.b: Why (DETRAN data) is it compared against Rt?

Change #1.8

Based on the disease characteristics, we expected that a change in the dynamics of human mobility would impact the infection rate after a given period of time. In other words, changes in mobility do not immediately affect the infection rate. As seen in Fig 9.b, as the reproduction number decreases, the normalized value of the daily trajectory frequency presents an increasing behavior. To confirm this relationship, we performed cross-correlation tests, where we found a maximum correlation when $lag = -1$. With the Granger causality analysis we confirmed that the vehicle flow "granger causes " the reproduction number $R(t)$ delayed by 1 day, that is, this result showed us that even with the realignment temporal data, we have to correct the DETRAN-CE vehicle flow data in one unit.

We modified the following paragraph in Section **Case study II: Fine-grained analysis** on Page 14:

> *"Due to the characteristics of the analyzed disease, we expected that a change in human mobility dynamics impacts the infection rate with a specific time lag. In other words, changes in mobility do not immediately impact the infection rate. As seen in Fig 9, as the reproduction number decreases, the normalized value of the daily trajectory frequency presents an increasing behavior. We can verify this relationship through cross-correlation tests, where we find a maximum correlation when $lag = -1$, as can be seen in Fig 8. "*

Comment #1.9

Fig 10: I guess that "values to be predicted" means "predicted values". Also, the curve looks like a simple exponential or a power law. How does the model prediction would compare with a simple exponential fit for instance?

Change #1.9

We corrected the legend accordingly. We agree with the reviewer that a simple exponential, power law regression, or another model that does not account for the mobility factor q_t would yield a good fitness. However, in this work, our focus is not on improving the model's performance but rather on obtaining more information about the system's dynamics. With the addition of mobility information and consequently the estimation of q_t, we break down uncertainty terms and capture the influence of population mobility. Consequently, we were able to answer new questions, which we were unable to answer with the standard SEIR model or with a simple exponential or power-law regression. For example, whether a given mobility intervention would have any effect. In addition, we also want to quantify this effect. Moreover, a simple exponential or a power-law regression will not capture the compartmental behavior, e.g., when the number of infected people is high, the infection tends to decrease intensity due to the corresponding decrease of susceptible individuals. We added this discussion in the text in Section **SENUR model equipped with mobility information** on Page 8:

> *"Although SENUR models without mobility can satisfactorily model the pandemic behavior, they cannot answer questions regarding the impact of mobility. For instance, we cannot quantify how the government measures of mobility restriction impact on the infection rate – Fig 10(a)."*

Comment #1.10

Fig 11(all): Why Rt stops at 5? Where does it go?

Change #1.10

We tried to enhance the visualization of specific regions. We agree that it can cause misinterpretation and changed the interval to accommodate all observed values of $R(t)$.

Comment #1.11

Fig 12(all): Again, I don't see we the lines are discontinuous. Also, what are the units on why axes? Seems very odd that the number of case reported peaks at 10 (Fig.12a).

Change #1.11

Fig 12 corresponds to the ratio between the result of our model when considering the Brazilian Health Ministry datasets and the following scenarios: I) the population did not change its mobility behavior and the government did not implement any restrictive measures and II) authorities decreed the closure of trade soon after the announcement of the first case of COVID-19. Our objective is to analyze the impact of mobility on the spread of the virus, comparing the real scenario with simulations of two extreme hypothetical scenarios. The peak at 10 indicates that whether nothing had changed and people had continued to behave like no pandemic was in course, we would have 10 times more cases than the result of our model when considering the Brazilian Health Ministry datasets.

To improve plots readability, we modified the ordinate axes caption to clarify that we are applying the ratio between model results when applying real data and when simulating these scenarios (Section **Case study II: Fine-grained analysis** , page 17).

Comment #2

Line 27: Avoid using the phrasing "critical transition" if it cannot be formally justified. Critical transitions are well defined in statistical physics and need to satisfy particular properties which I don't think apply to the case study.

Change #2

We thank the reviewer for raising this point. We rephrased our text to cut the use of this term (Page 2):

> *"The restriction, alongside measures of social distancing and quarantine, has rapidly decreased the force of infection and hence controlled the disease spread.* ***Using human mobility data, authors observed this change immediately after an intervention by measuring the correlation between the mobility indexes and the growth rate of the disease."***

Comment #3

Eq.(1-5) is a technically a system of Ordinary Differential Equations (ODEs), not Partial Differential Equations (PDEs). PDE are usually invoked when dealing with multivariable functions. On the other hand, in this case each compartment is only a function of time.

Change #3

We thank the reviewer for remarking that. We changed the text accordingly in Section **SENUR model equipped with mobility information** on page 7.

Comment #4

Infected sub-compartments are presented as I_{N_i} and I_{U_i}, but in the equations appear I_{C_i} ans I_{S_i}, which are not defined. What are these compartments? How they relate to the previous ones?

Change #4

Actually we have only I_{N_i} and I_{U_I}, which were wrongly introduced as I_{C_i} and I_{S_I}, respectively. We made these corrections in the manuscript.

Comment #5

Why should d_N and d_U be different?

Change #5

To generalize the model, we adopt d_N and d_U as distinct values associated with different types of infected individuals (notified and unreported). This difference between d_N and d_U is assumed because reported/unreported individuals have different dynamics, as already observed in some works in the literature. We modified the Section **SENUR model equipped with mobility information** on Page 7:

> *" where d_E^{-1} represents the transition rate from exposed individuals $E_i(t)$ to infected individuals $I_{N_i}(t)$ and $I_{U_i}(t)$; d_N^{-1} represents the transition rate from notified infected individuals $I_{N_i}(t)$ to removed individuals $RE_i(t)$; and d_U^{-1} represents the transition rate from underreported infected individuals $I_{U_i}(t)$ to the removed individuals $RE_i(t)$, **in concordance with [18, 28, 29]**. "*

Comment #6

In the expression for λ_i a μ appears, not defined. Please, clarify what μ is.

Change #6

We added in the text the μ_i definition in Section **SENUR model equipped with mobility information** (Page 8). It now reads:

> *"where μ_i is a regularization term that defines how nonpharmaceutical interventions (NPIs) affect the virus transmissibility rate in the cluster i. For instance, if the population uses masks and applies other NPI measures, the μ_i must decrease."*

Since the regularization term is specific to each region i, the μ parameter was incorrectly added in the text. We removed it from the revised manuscript.

Comment #7

What is an internal mobility index?

Change #7

We intended to say that our matrices were converted to an index when using only the region. When using the mobility index, we consider all the paths of individuals in the analyzed region, regardless of their origin or destination (coarser spatial granularity). However, to make our text more readable, we decided to change the text as follows (Section **SENUR model equipped with mobility information**, Page 8):

> *"This model can also be applied to an index when using only the region. When using the mobility index, we consider all the paths of individuals in the analyzed region, regardless of their origin or destination (coarser spatial granularity). So, we can simplifies the force of infection equation to:"*

$$\lambda(t) = \mu \times W(t) \times \left(\frac{I_N(t) + I_U(t)}{N} \right)$$

Comment #8

What are the possible values for q_i in general?

Change #8

q_i is the mobility index introduced by us in this work. We defined $q_i \in [0, 1]$ so that it can be compared among different scenarios.

We defined q_i possible values in section **SENUR model equipped with mobility information** on page 7 and it now reads:

> *"The impact of mobility on the virus spread at time t is given by the matrix $W_{ij}(t) = q_t C_{ij}$, where $q_t \in [0, 1]$ is a scalar, time-dependent parameter estimated by the model to quantify the influence of mobility on pandemic dynamics."*

Comment #9

Line 336: 2785% with respect to? Same apply for every other percentage. Please clarify the meaning of every percentage.

Change #9

These percentages are related to the ratio of the the number of infected people estimated by our model (null model – model output estimation from real data) and the number of infected people estimated by the model considering the scenarios related to the experiments **Scenario I** and **II**.

For instance, considering the **Scenario I**, we verified that if the government had not taken any restrictive measure, our model indicates a 27.85-fold increase in the number of notified infected people (in Fortaleza) when compared to the null model.

To clarify this experiment, we reword the manuscript as in Section **Case study I: Coarse-grained analysis** on Page 11:

> *In these experiments, we analyze the ratio between the number of infected individuals under a given scenario and the number of infected individuals estimated by our model. Ratios smaller (resp. greater) than 1 represent a decrease (resp. increase) in the number of infected individuals under the hypothetical scenario relative to what took place in reality.*
>
> *Scenario I resulted in a steep increase in the number of infected individuals of 27.85 times for Fortaleza (Fig 6.a), 75.62 times for Rio de Janeiro (Fig 6.c) and 66.31 times for São Paulo (Fig 6.e) at the end of the analyzed period, relative to the actual numbers at that same point in time.*

Comment #10

The English overall can be largely improved.

Change #10

Thanks, we revised the paper and fixed all language issues we could find.

III. Reviewer #2

Comment #1

In the abstract, it is stated that "This work is the first to shed light on the pandemic situation on the Brazilian territory using both aggregated (...) and fine-grained (...) mobility data (...)": It is not true. Here I cite some works that investigated this issue in Brazil using these kinds of data:

- "Assessing the potential impact of COVID-19 in Brazil: mobility, morbidity and the burden on the health care system." medRxiv 2020.03.19.20039131 (2020)
- "Evolution and epidemic spread of SARS-CoV-2 in Brazil." Science 369.6508 (2020): 1255-1260.
- "Modeling future spread of infections via mobile geolocation data and population dynamics. An application to COVID-19 in Brazil." PLOS ONE 15.7 (2020): e0235732.
- "Outbreak diversity in epidemic waves propagating through distinct geographical scales." Physical Review Research 2(4) (2020): 043306.
- "Spatiotemporal pattern of COVID-19 spread in Brazil." Science 372.6544 (2021): 821-826.

None of these works are on the reference list.

Change #1

Thanks for pointing this out, we actually intended to express that our work investigated both coarse and fine granularity, differently from previous work. To avoid misleading the reader, we removed part of the sentence that does not clearly state our thoughts.

Additionally, we thank the reviewer for providing additional relevant references to enrich our work. We added all of them in the Introduction Section.

Comment #2

The mobility restrictions seem to be a change in the infection rate, as a "social distancing" measure or other NPIs. If the authors had mobility data as a function of time (that was not clear to me), such as the data available in:

- "Heterogeneous impact of a lockdown on inter-municipality mobility." Physical Review Research 3.1 (2021): 013032.
- "COVID-19 lockdown induces disease-mitigating structural changes in mobility networks." Proceedings of the National Academy of Sciences 117.52 (2020): 32883-32890.

it would be interesting. However, the model is based on a parameter q_t that is calibrated and no sensitivity or calibration analysis was presented to show the possible correlation between different parameters. I am not convinced that the mobility data used were important to draw conclusions.

Change #2

We agree with the reviewer, and appreciate the considerations.

In the current manuscript, we added a complete new section (**Sensitivity analysis**, Page 17) providing a sensitivity analysis of the calibration of parameter q_t.

Sensitivity analysis

In this work, we estimate a mobility factor q_t from our model to quantify the mobility dynamics of the COVID-19 pandemic in some Brazilian cities. In order to assess the sensitivity of our proposal, we compared our mobility quantifier with mobility data series used in our work (Waze report and DETRAN-CE dataset), as shown in Fig 13.

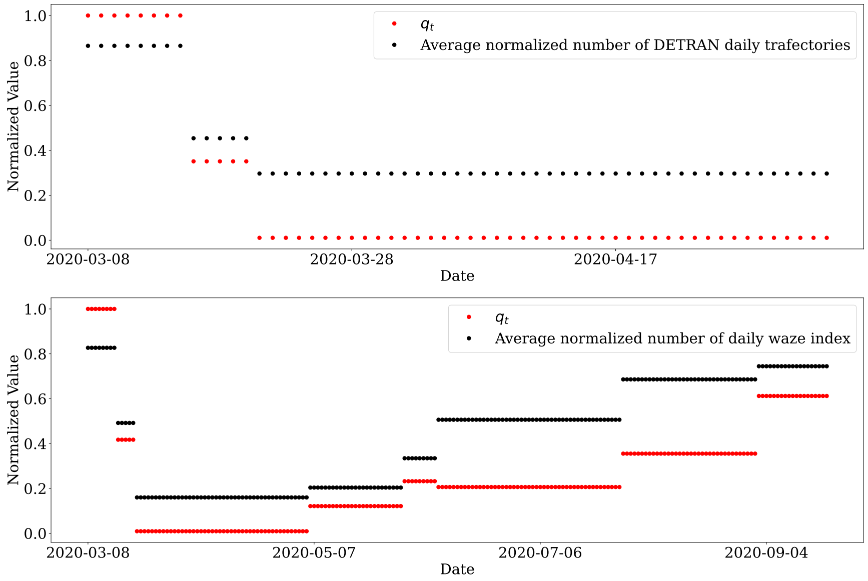

Fig. 13. **Sensitivity analysis.** Comparison between our mobility quantifier with some real mobility data used in our work.

Initially, we compared q_t *with the flow of vehicles in the city of Fortaleza. Then, we compared the estimated values of* q_t *in section* ***Case study II: Fine-grained analysis****, with the periods analyzed at the average normalized number of DETRAN-CE daily trajectories. We observed the trend between these two components, where in the first period* $q_0 = 1$*, we have the value of* 0.8651 *found in the normalized mean series of the vehicle flow, which is the highest value among the analyzed periods. As the mobility index value decreases, as we can see in* $q_1 = 0.351$ *and* $q_2 = 0.0106$*, we find that the average normalized of the vehicle flow also decreases to* 0.4537 *and* 0.2966*, respectively. We also analyzed the case with coarse granularity. In this case, we compared the value of* q_t *with the normalized mean of the Waze mobility index. In this case, we observe the same trend, i.e., excluding the period 06/08 to 07/27 (*q_5*), as the value of* q_t *increases/decreases, the value of the normalized mobility index of the Waze increases/decreases respectively. Furthermore, it is worth noting that for the period used in the two figures, the values of* q_t *are consistent; that is, for the case of fine granularity, we find* $q_1 = 0.371$ *and* $q_2 = 0.0106$ *while for the case of coarse granularity we have* $q_1 = 0.417$ *and* $q_2 = 0.009$*,*

indicating that our technique estimates similar values for the same period, even using different modeling and data sources.

It is worth noting that the q_t is a parameter estimated by our model. In our analysis, we estimated q_t every time a mobility restriction/release was announced, however, q_t can be estimated periodically in the case it is more adequate. For instance, q_t can be estimated daily, weekly or biweekly, similarly to the usual $R(t)$ estimation.

Comment #3

No discussion was made about each city's results, of how the measures adopted there were efficient or not to mitigate the spread. There is only a discussion about how the numbers would change if different strategies were adopted.

Change #3

We agree that a broader discussion of how adopted measures should mitigate or not the virus spread would enrich our main results. However, this analysis is very hard to cope, perhaps leading to misleading conclusions, once we do not have data to perform any causal inference about the real effectiveness of mobility measures to restrict the spread of the virus.

In scenario simulations, we compared the proportion of infected individuals from an intervention measure to our model due to the observational nature of our data. Still, we did not infer any causal effect from this study.

We added this data to Table 1 (q_t). To show that q_t is a reasonable indicator of mobility. The reader can observe that it captures the mobility restrictions impact imposed by governments, as shown in Table 1.

We added in Section **Case study I: Coarse-grained analysis** on page 10:

Table 1 shows how we assign q_t to different periods in all cities studied.

For instance, for Fortaleza, q_0 corresponds to the period before FO_0 (the first notified case), q_1 to the period before FO_1 (trade closure), and so forth. Initially, we noticed that all cities analyzed had reduced mobility with the appearance of the first quoted case of COVID-19. This behavior is expected because people naturally tend to reduce mobility due to the fear of contagion after the appearance of the first notified case. Furthermore, we observe that São Paulo has the highest q_1 mean, probably because it was the first

case reported in Brazil.

We observed some general behaviors for the cities analyzed. For example, after adopting trade closure measures, the decreased q_t *average, thus showing evidence of the effectiveness of this measure. It is worth noting that we do not have counterfactual data, i.e., how would the pandemic behave if the trade was not closed? We only have evidences (in this case, its correlation) of the effectiveness of the measures. We are not able to make any causal inference to state that the trade closure indeed yields to a decrease of the pandemic.*

We also observe some interesting individual behaviors. Porto Alegre had two periods of intervention. After the first, we noticed that in the reopening of civil construction activities, the city had a $q_4 = 0.423$*. However, after the second moment, the intervention measure proved to be much more effective, indicating* $q_7 = 0.121$*, corresponding to the period of closing of parks and beaches. Furthermore, we see that Porto Alegre has the lowest mobility index value at the end of our analysis (*$q_9 = 0.237$*). This number combined with the fact that Porto Alegre was heavily affected by the H1N1 previous pandemic suggests that the population may have been more alert to the new pandemic (SARS-COV2), and thus, reacted earlier.*

The cities of São Paulo and Rio de Janeiro reopened bars and restaurants and we observed an increase of q_t *after the adoption of these measures.*

Comment #4

Figures: Each figure uses a different style and notation. Not even the legends of the figures were adapted to fit the space, and in some cases the legend hides the curves or points.

Change #4

Following the reviewer suggestions, we replotted our graphs to improve the results readability.

Comment #5

Abstract) What the authors mean by "data from public sources"? The data is not available publicly and this phrase sounds like it is.

Change #5

The data is extracted from public services, although they are not available due to privacy concerns. To avoid misunderstandings, we changed the abstract text to:

> *"Using both aggregated (from large Internet companies) and fine-grained (from Departments of Motor Vehicles) mobility data sources, our work sheds light on the effect of mobility on the pandemic situation in the Brazilian territory."*

Comment #6

p.1) "The negative effects of the COVID-19 pandemic in Brazil may be related to the lack of knowledge about the disease and virus characteristics, such as its lethality and high transmissibility": I do not agree with the "lack of knowledge", it may be something else.

Change #6

Reading the sentence again, we agree with the point raised by the reviewer. We thus dropped the sentence.

Comment #7

p.2) It is important to distinguish two types of mobility: the flow of people between different areas (such as neighborhoods) and inside each of these areas. At the beginning of the epidemics, interventions of mobility between countries, or even municipalities are important to mitigate the spread from one place to the other by avoiding the mixing of people. Now, however, with cases confirmed in all municipalities and a high number of new cases and deaths every day, it is more important to use a social distancing approach and others NPIs, such as masks to reduce the level of contagion. The mobility can drive the spatio-temporal pattern, but other factors are more important to the local spread. In the way the mobility data was introduced in the model, it seems to be only related to the local spread.

Change #7

We fully agree with the comment made by the reviewer. Actually, given the data we have, our analyses focus on the intra-municipality regions, which are all driven by the local administration policies w.r.t. actions that prevent and mitigate the virus spread. To make clear this point, we

rephrased the following paragraph (Section **SENUR model equipped with mobility information**, Page 8).

> *"The granularity of the resulting model is directly associated with the data granularity. Thus, if we only have mobility information about one region, we can only use our model to make inferences about this single region.* ***Otherwise, if the mobility data contain information that break downs the region into smaller areas (e.g., neighborhoods in a city), we can make inferences at a finer granularity."***

Comment #8, #9, #10

p.3) Lines 86-88: "shows" -> "show", "we concludes" -> "we conclude", "and discusses" -> "and discuss".

p.3-4, and in the rest of the paper): Please choose a date format and keep that throughout the text. In Page 3, the MM/DD/YYYY is used, then in line 135 the format MM/DD/YYYY is used together with DD/MM/YYYY (probably a typo). Later, in Fig 1c, MM/DD/YYYY is used, but in Figs 5(a-e), 6(a-f) and 10 the YYYY-MM-DD format is used.

p.4) Is the quotation in lines 118-120 really necessary for the paper?

Change #8, #9, #10

We made all the corrections suggested by the reviewer.

Comment #11

p.5) The "Coronavirus Panel data" contains the number of cases and deaths by confirmation or report date, not the notification date as in "Opendata SUS". The first is affected by delays related to inserting the record in the system, the exam collection date, the exam result date, and finally the reporting from the municipality to the state's health department. So, the methodology used by Abbott et al is not enough, in this case, to "rewind" the data by using only the delays from symptoms onset to the notification. It is clear to me when I see Fig 1(c) and 1(d): the peak around September 2020 appears as a sudden drop in Fig 1(d). The peak is related to delays in confirming the cases, not in notifying the cases in the system (when the patient seeks medical attention), as the authors correctly stated in lines 178-179. However, I am not convinced that an adequate

methodology was used in this case. Please see other methodologies to correct reporting delays, such as
- "A modelling approach for correcting reporting delays in disease surveillance data." Statistics in Medicine 38.22 (2019): 4363-4377.

Change #11

We appreciate the comment as well as the excellent observation. In this work, additionally to the methodology proposed by Abbott et al., we also used a correction in ‘rewind’ method. Observe that in the ‘**Case study II: Fine-grained analysis**’ section, we analyzed the lag by observing the correlation between $R(t)$ and cars flow (Fig 8).
We obtain lag = -1; this value is applied to correct the time difference between the series and thus realign the data. Since we do not have information regarding the delays to inserting the record in the system, we believe that the additional methodology we applied here (the correlation lag) corrects the delay adequately. We clarified this information in the revised manuscript (Section **Case study II: Fine-grained analysis**, page 15).

> *We were able to verify that the past of the vehicle flow, delayed* 1 *day, helps to predict the present value of* $R(t)$ *of COVID-19, indicating a causal sense among them. So, the flow of vehicles "Granger causes" the reproduction number* $R(t)$ *delayed by* 1 *day, i.e., even after the temporal re-alignment of the data, we have to correct DETRAN-CE's vehicle flow data in one unit.*

Comment #12

Fig 1: there are no labels in Figs 1(a,b). What do the y-axis and x-axis mean? In Fig 1(b), what does the dashed line mean? Why (a) and (b) are in different plots, if (a) is the empirical distribution of the delays and (b) the estimated one? Should not the bars be the empirical distribution and the curve the estimated one? Please clarify.

Change #12

We changed the axes of the figures:
Thank you for the thoughtful correction. The x and y axes of Figs 1 (a, b) correspond respectively to the delay values and the probability of their occurrence. The vertical lines refer to the average delay value present in each of the distributions.

Such graphics correspond to:

(a) Empirical delay distribution taken directly from the OpenData SUS dataset and the mean of this value (represented by the vertical line),

(b) Theoretical delay distribution, obtained by fitting the empirical distribution as a gamma or exponential distribution (depending on the analyzed dataset) and again the mean of this value.

Thus, we chose to view these two distributions separately to highlight the quality of the fit applied.

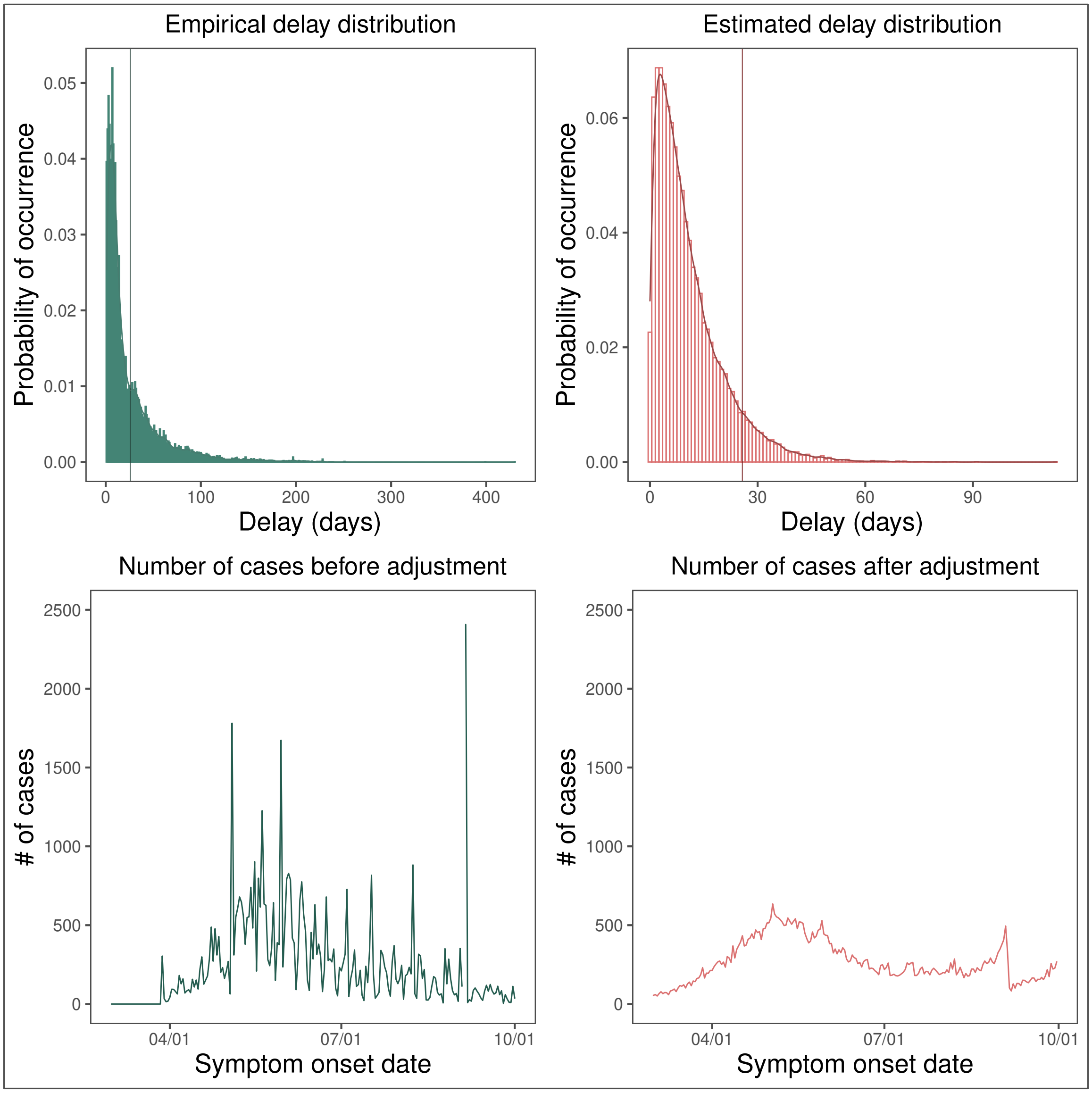

Comment #13

p.5, 6, and so on) Another problem with the notation appears in the manuscript: I_{N_i} and I_{U_i} are used in p. 5, but in Eqs. (1-5) they appear as I_{C_i} and I_{S_i}, and I_{C_i} in line 266 (p. 8). Please clarify.

Change #13

We unified the notation to I_{N_i} and I_{U_i}.

Comment #14

p.7) μ_i and μ are not defined.

Change #14

We refer the reviewer to our response to comment #1 of reviewer #1 where we defined μ_i.

Comment #15

p.7) "Alternatively, if this information is associated as a function of smaller regions, we can make inferences with finer granularity." What does that mean?

Change #15

We refer the reviewer to our response to comment #7 of reviewer #2.

Comment #16

p.7) Notation problem: here "R_t" is used, but on Page 13 it appears as $R(t)$ (and caption of Fig 8)

Change #16

We agree and modified it in the text to avoid this notation problem. We unified it to $R(t)$.

Comment #17

p.7) The value of q_t is calibrated in $W_{ij}(t) = q_t C_{ij}$ to find the values of q_t that better represent the situation in a given time window, correct? The problem is that as μ_i is a free parameter (and not defined), anything can "fit" the real data. Also, it is not clear if C_{ij} is constant in time.

Change #17

We agree with the reviewer's comment, and apologize for the confusion.

Indeed, q_t is calibrated to better represent the situation in a given time window.

We corrected the definition of μ, as seen in *Comment #18*. Our actual model considers μ_i as a multiplicative instead of a additive term. It was misleading in the original text. Therefore, the observation that μ_i is a free parameter and fits "anything" about the real data does not hold for this model. We apology for this confusion as our first approach was using μ as an additive term, but we observed the problem you have pointed out. We updated the model but mistakenly forgot to update the text.

In this work, we are using two datasets: Opendata SUS and Coronavirus Panel data. The Opendata SUS dataset has more detailed information about the cases and the location of the patients. In contrast, Coronavirus Panel data has information about the behavior of the pandemic (infected, recovered, and the like), but we do not have individual information.

For the experiment with fine granularity, we use the Coronavirus Panel data to estimate all parameters. However, for the calibration/sensitivity of the term q_i, we also use information from Opendata SUS. In our model, q_t captures information about mobility, while μ models the virus transmissibility rate.

Note that, to calibrate the values of regions with fine granularity, we use Opendata SUS. Therefore, we consider the ratio $I_{N_i}(t)/I_{N_j}(t)$ for calibration/sensitivity of the $q_i(t)$ term, where $I_{N_j}(t)$ is the number of infected individuals notified from the region with the lowest number of cases for a day t. In other words, our model estimates the parameters using the Opendata SUS dataset so that its prediction keeps the ratio observed in the Coronavirus Panel data. Furthermore, to avoid small sample issues, we consider for calibration only the days where $I_{N_j}(t) > 10$.

Regarding the C_{ij} term, it is estimated for every time window, so, it is constant only at a given time window.

We add the paragraph to the following (Section **Case study II: Fine-grained analysis**, page 13):

> *Note that, to calibrate the values of regions with fine granularity, we use Opendata*

SUS. Therefore, we consider the ratio $I_{N_i}(t)/I_{N_j}(t)$ for calibration/sensitivity of the $q_i(t)$ term, where $I_{N_j}(t)$ is the number of infected individuals notified from the region with the lowest number of cases for a day t. In other words, our model estimates the parameters using the Opendata SUS dataset so that its prediction keeps the ratio observed in the Coronavirus Panel data. Furthermore, to avoid small sample issues, we consider for calibration only the days where $I_{N_j}(t) > 10$.

Comment #18

p.7) The force of infection is $\sum_j \left[W_{ij}(t)\left(\frac{I_{N_i}(t)+I_{U_i}(t)}{N_j}\right)\right]$, meaning that infected individuals from i interact with N_j individuals in j. What is the explanation for that choice?

See Sec. 7.2 of:

- Keeling, Matt J., and Pejman Rohani. Modeling infectious diseases in humans and animals. Princeton university press, 2011.

Change #18

We agree with the reviewer's comment, and apologize for the confusion. Our actual model considers μ_i as a multiplicative instead of a additive term. Besides, we mistakenly used index i in the numerator and j in the denominator. We fixed the equation as follows (Section **SENUR model equipped with mobility information**, page 8):

Our infection strength is defined as:

$$\lambda_i(t) = \mu_i \times \sum_j \left[W_{ij}(t)\left(\frac{I_{N_j}(t)+I_{U_j}(t)}{N_i}\right)\right],$$

where μ_i is a regularization term that defines how nonpharmaceutical interventions (NPIs) affect the virus transmissibility rate in the cluster i, i.e., μ_i models the actual virus transmissibility rate between the susceptible individuals from i (N_i) and the infected individuals from j ($I_{N_j}(t) + I_{U_j}(t)$).

Comment #19

p.7) In Eqs. (1-2) what is the meaning of the factor $I_{C_i}(t)/N_i$? Has this not already been counted in the infection force? Also, in Eqs. (1-2) it shows $\lambda_{i,t}$, and later as $\lambda_i(t)$.

Change #19

We agree with the reviewer's comment, and apologize for the confusion. As we stated in *Comment #18*, the model description in the original submission was outdated. All results we presented used the correct model, i.e., with μ as a multiplicative term. We used as reference the paper "Age-dependent effects in the transmission and control of COVID-19 epidemics"[a], which also uses a multiplicative term, so that, the model dynamics also changes to:
In fact, our model dynamics is defined as (Section **SENUR model equipped with mobility information**, page 7):

$$\frac{dS_i(t)}{dt} = -\lambda_i(t)S_i(t), \quad (1)$$

$$\frac{dE_i(t)}{dt} = \lambda_i(t)S_i(t) - y_i(t)d_E^{-1}E_i(t) - [1 - y_i(t)]d_E^{-1}E_i(t). \quad (2)$$

[a]Davies NG, Klepac P, Liu Y, Prem K, Jit M, Pearson CAB, et al. Age-dependent effects in the transmission and control of COVID-19 epidemics. Nature Medicine.6472020;26(8):1205–1211. doi:10.1038/s41591-020-0962-9.

Comment #20

p.8) There is no information on the number of cases for each region, correct? I mean, the number of cases is only available at the city level, not by the regions.

Change #20

As commented in our answer to *Comment #17*, of the two datasets used, Opendata SUS has information about the regions (however, this dataset is much smaller when compared to Coronavirus panel data). Therefore, we have information about the infected in regions but with much fewer data.

Text Modifications (same of Change #31) in Section **Data sources** on page 4:

> *"Opendata SUS [21], a smaller dataset that includes more details on clinical (e.g., the date of the first symptom presented by the patient and your case evolution) and*

demographic (e.g., the patient's residence) information of the cases.

. . .

The analysis of the COVID-19 evolution in relation to the human development index (HDI) used data obtained by the study carried out by the Fortaleza Municipal Secretariat for Economic Development (SDE) [22]. These results used data from the last Brazil Demographic Census carried out in 2010 as a basis."

Comment #21

p. 9, Table 1) What are the values of q_t for each case in Table 1? Maybe the median or mean value can be put in the table. The caption text does not describe anything and should be changed. Also, from where were these dates extracted for each city?

Change #21

We added q_t to Table 1 in Section **Case study I: Coarse-grained analysis** on page 10 and we changed the caption to the following:

> *"**Periods used to estimate the mobility parameter** q_t **for different cities.** The table presents the periods used in our model to estimate the different values of the mobility parameter q_t alongside the q_t sample mean. We collected these periods from news provided by the government of each city in 2020. The description describes the main events related to the period."*

Comment #22

p.9, line 298) There is a typo: "q_1 e q_2" -> "q_1 and q_2".

Change #22

We agree and modified it the text. We corrected other typographical and grammatical errors as well.

Comment #23

p.10, Fig 4) The caption says that q_1 and q_2 are being shown, but in Fig 5(b) we have q_3. Please clarify.

Change #23

We corrected the legends of the Fig 4 in section **Case study I: Coarse-grained analysis** on page 11, so, for the cities of Fortaleza and Rio de Janeiro the period related to the trade closing consists of q_2 and for São Paulo q_4.

Comment #24

p.10) Were the results compared to a null model discarding the mobility data? I think it is important to state that the mobility data is necessary for the calibration. A calibration or sensitivity analysis is necessary to draw any statistical conclusion. My feeling is that the mobility is not playing a role here: a SEIR model with a time-dependent rate (related to the q_t parameter in this case, and the dates in Table 1), without mobility data whatsoever, can be enough. See

- "A SEIR-like model with a time-dependent contagion factor describes the dynamics of the Covid-19 pandemic." medRxiv 2020.08.06.20169557 (2020)

Change #24

We agree that time-dependent rate models without mobility data (e.g., SEIR) can satisfactorily model the pandemic behavior. However, these models cannot be used to make inferences about the effect of changes in mobility. Therefore, the inclusion of mobility data in the model was not motivated by a quantitative increase in the model's accuracy. Instead, we add this information to quantify the city's mobility parameters, and consequently, answer questions that the SEIR model alone would not be able to respond (for example, quantify how the government measures of mobility restriction impact on the infection rate).

In addition, we compared our result with the null model discarding the mobility data, as we can see below (the black line indicates the null model's prediction):

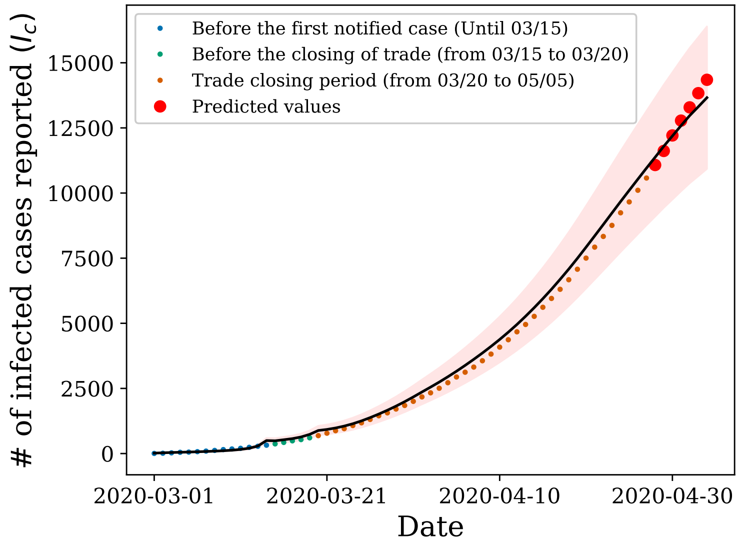

We see that the null model fits to the data similarly with our model; however, with this model, we cannot get estimates of mobility associated with $q_i(t)$, thus making it difficult to answer the questions associated with mobility dynamics.

We added this information in the text to make it clear. Now it reads (Section **Case study II: Fine-grained analysis**, page 15):

"Although SENUR model without mobility can satisfactorily capture the pandemic behavior, it cannot answer questions regarding the impact of mobility. For instance, we cannot quantify how the government measures of mobility restriction impact on the infection rate. Therefore, we propose in this work an extension of this model to investigate human mobility's influence on the spread of the COVID-19 pandemic. We can observe in Fig 10 (a) that the confidence interval is wider than the one presented in Fig 10 (b). It is an evidence that our model captures part of the variability of the model by estimating the mobility. Therefore, although both models can be satisfactorily used, our model is more expressive in terms of mobility and it is more accurate for the analyzed data. "

Comment #25

p.10, Fig 5 and simulation results) The confidence interval is very weird. How do the authors explain these piece-wise confidence intervals? Were the data calibrated for each time window with a constant q_t, but a different set of other parameters? If so, that does not seem to be correct,

since these time windows are not independent. See Fig 5(c), for example. Why not plotting also the median or average value of the simulations, instead of only the weird confidence intervals?

Change #25

We appreciate the comment. We fixed the confidence intervals and added the mean to the plots. Time windows have a time dependency between analyzed periods. Therefore, for each simulation, we consider as initial state ($S(t_0)$, $E_i(t_0)$, $I_{N_i}(t_0)$, $I_{U_i}(t_0)$ and $RE_i(t_0)$), the values obtained by the model in the last day. We perform this restriction to consider the time dependence between simulations.

We add the estimated average value q_t for each period to Table 1 and we plot the average value in model's prediction.

Comment #26

p.11, Fig 6) The y-axis label seems to be wrong. For q_0, the value is close to 1. Is it the ratio between the number of infected cases reported using this strategy, compared to the calibrated one, or the $\%$? In the caption, it says that it is the ratio, but uses "$\%$" in the label. Please clarify.

Change #26

Thank you for the correction. We fixed the text of the y-axis label, changing it to **'Ratio of'**.

Comment #27

p.11 Fig 6b) If it is the ratio, why are the values different of 1 when using q_0? What set of parameters was used in this case? Is it a fixed one, since no confidence interval is present?

Change #27

Initially, at the beginning of the pandemic, the number of infected is relative small. Thus, even using the same set of parameters for q_0, due to the stochastic variability of the simulation, the ratio is not exactly 1, but values close to 1 (e.g., the city of Fortaleza has about 100 infected individuals during this period analyzed, so a variation of 5 notified individuals corresponds to 0.05 difference from the initial value). We changed and improve this plot to force the ratio to 1 to avoid confusion.

Therefore, for each period used in scenarios I and II, the average value associated with the parameters estimated in each scenario was used, thus justifying the absence of confidence intervals. We changed it in the text as indicated (Section **Case study I: Coarse-grained analysis** , page 12):

> *" Initially, at the beginning of the pandemic, the number of infected is tiny. Thus, even using the same set of parameters for q_0, the ratio is exactly 1. "*

Comment #28

p.11 Fig 6) Why some figures have q_0 and q_2, and others only q_0 in the legends? Please clarify.

Change #28

We refer the reviewer to our response to comment #1.4 of reviewer #1.

Comment #29

p.11) What the authors mean by "although it is likely to happen" in line 352? What about the new waves of infection with the new variants and mutations?

Change #29

Our work does not consider new variants, mutations, or waves. We changed the text to make it clear as follows (Section **Case study I: Coarse-grained analysis**, page 12):

> *"It is also worth noting that although the model output indicates that the pandemic would be at its ending by May 2020, even without adopting mobility restriction measures, this would cost millions of deaths and a total collapse of the health system, as the number of infections would rise dramatically. This result does not consider new variants, mutations, or new waves of infection, which could prolong the pandemic."*

Comment #30

p.11) "HDI" is not defined here, only on the next page.

Change #30

We changed it in the text as indicated. Now we defined HDI in Section **Data sources** on Page 5:

> *"We base the construction of these sub-regions on geographic connectivity and the human development index (HDI)."*

Comment #31

p.12 and Fig 7) It was not clear to me if the number of infected people in each region of Fortaleza was obtained from the simulations or if they are real data. If so, what is the source of this data? If it is official reporting data, it does not seems to be related to any mobility data mentioned in the main text.

Change #31

First, we would like to thank you for your thoughtful comment. Data related to the evolution of cases regarding cure, infection permanence, or home treatment were taken directly from the Opendata SUS dataset. In fact, this data is not related to any mobility data.

The information related to the human development index was obtained based on a study by neighborhood carried out by the Fortaleza city in 2014.

Text Modifications (same of Change #20):

> *"Opendata SUS [21], a smaller dataset that includes more details on clinical (e.g., the date of the first symptom presented by the patient and your case evolution) and demographic (e.g., the patient's residence) information of the cases.*
>
> ...
>
> *The analysis of the COVID-19 evolution in relation to the human development index (HDI) used data obtained by the study carried out by the Fortaleza Municipal Secretariat for Economic Development (SDE) [22]. These results used data from the last Brazil Demographic Census carried out in 2010 as a basis."*
>
> ...
>
> *The social clusters applied in this work were built based on two local characteristics: geographic connectivity, and the HDI.*

Comment #32

p.13) The analysis of the correlation between DETRAN-CE and Google Mobility data is very interesting, especially the lag between $R(t)$ and the time series.

Comment #32

Thank you for the comment.

Comment #33

p.14) I understand that "using a regional approach, we can stratify the information for each region individually". In the caption of Fig 11, the authors state the number of infected cases was estimated by their model. Without comparison to official reporting data aggregated by region rather than the city as a whole, we cannot conclude anything. If the mobility data is used in a model, it must be compared to official data, or with a null model to show that this was really necessary. Also, again I stress the need for sensitivity analysis of the calibrated parameters.

Comment #32

We appreciate the reviewer's considerations, and have added a section on the sensitivity analysis as described in *Comment #2* and *Comment #24*.

Comment #34

p. 14, Fig 11) Why is Rt so large close to 04/06? No discussion was made about this fact.

Change #34

We are grateful for the observation regarding this fact. After an investigation, we observed that Fortaleza started at alarming rates about the pandemic. However, the population's perception of Covid-19 changed after the death of a 3-month-old baby in the state (on 03/04)[a].

Text added (Section **Case study II: Fine-grained analysis**, page 16):

> *"We can see the peak in the graphs in Fig 11. After an investigation, we observed that*

Fortaleza changed the perception of Covid-19 after the death of a 3-month-old baby (on 03/04).

On 04/04, the state government released a statement about this fact and reinforced the pandemic's seriousness. The great commotion is likely to be related to the steep drop in the value of the observed $R(t)$."

[a]https://www.opovo.com.br/coronavirus/2020/04/06/crianca-de-tres-meses-de-idade-morre-em-iguatu-por-complicacoes-respiratorias-provocadas-pelo-coronavirus.html

Comment #35

p. 15) My feeling is that similar conclusions for all municipalities investigated could be obtained without using the mobility data. Instead of using W_{ij}, the authors could use, for example, the demographic density to estimate the number of contacts of each region, multiplied by q_t to infer the social distancing and others NPIs.

Change #35

We agree with the reviewer's comment. However, in this work, the contribution is to estimate q_t directly from the model, i.e., to present a solution that estimates the pandemic dynamics as well as the mobility quantifier directly from the model. Whith our model, we can simulate scenarios such as shown in Figs 6 and 12.

Comment #36

p. 15, Fig 12) Again, the caption says that it is the ratio, but the plot shows "# (Number) of". By the way, I suggest replacing all the "# of" with "Number of".

Change #36

We refer the reviewer to our response to *comment #26* of *reviewer #2*.
