# Supplementary material for: Effects of population mobility on the COVID-19 spread in Brazil
Source: PLoS One. 2021 Dec 7;16(12):e0260610. doi: 10.1371/journal.pone.0260610 (PMC8651143; doi:10.1371/journal.pone.0260610)
Supplement: Attachment — Submitted filename: Journal___PLOS_ONE-14.pdf [file pone.0260610.s003.pdf]

Response Letter of article PONE-D-21-13982: Effects of population mobility on the COVID-19 spread in Brazil

Eduarda T. C. Chagas, Pedro H. Barros, Isadora Cardoso-Pereira, Igor V. Ponte, Pablo Ximenes, Flávio Figueiredo, Fabrício Murai, Ana Paula Couto da Silva, Jussara M. Almeida, Antonio A. F. Loureiro, Heitor S. Ramos

I. Journal Requirements

Comment #1

Please review your reference list to ensure that it is complete and correct. If you have cited papers that have been retracted, please include the rationale for doing so in the manuscript text, or remove these references and replace them with relevant current references. Any changes to the reference list should be mentioned in the rebuttal letter that accompanies your revised manuscript. If you need to cite a retracted article, indicate the article's retracted status in the References list and also include a citation and full reference for the retraction notice.

First, we would like to thank the editor for handling this manuscript. In the light of the provided reviews, we have prepared a revised version of the manuscript. We would like to point out that all reviews were very important to strengthen our contribution and enrich the paper quality. We thank the reviewers for helping us in this process.

This response letter addresses all the comments in red, followed by our responses, and, whenever necessary, the changes made (in black). We also include the `diff` article between the prior and current versions, where deletions are in red, and additions are in blue.

Regarding our reference list, we have maintained all previously cited articles, as none have been retracted, and added the reference below:

Serafino M, Monteiro HS, Luo S, Reis SD, Igual C, Neto ASL, et al. Superspreading k-cores at the center of COVID-19 pandemic persistence. arXiv preprint arXiv:210308685. 2021.

II. Reviewer #1

Comment #1

If I have understood correctly, the model is fitted to the cumulative number of cases, however it is a more common practice to fit it to the daily incidences, which is also a much more insightful variable. With that I am not saying that the authors should do the fit again, but at least adding a couple of panels in order to see if the fitted model captures daily incidences as well would be definitely a nice add.

Change #1

Thank you for the careful comment. Our fitted model also captures daily incidences as shown in the following Figure. We added this Figure in Section **Case study I: Coarse-grained analysis**.

> *Note that, in Figure 5, our model estimates the accumulated value of infected for each city. Additionally, our model also fits quite well the number of daily cases, as illustrated in Figure 6 for the cities of Belo Horizonte and São Paulo.*

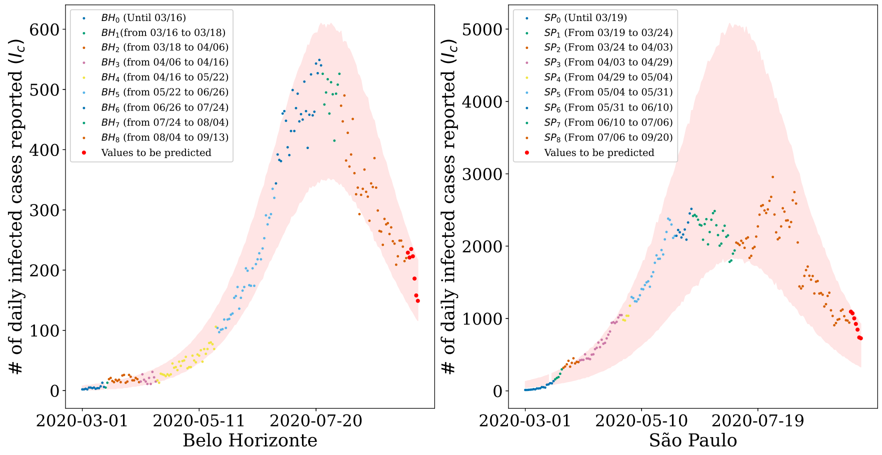

Fig. 6. **Simulation results for daily infected cases reported.** Number of infected people estimated by our model for the cities of (a) Belo Horizonte and (b) São Paulo. The shaded areas represent the 95 % confidence interval provided by the model and the points the official daily values released.

Comment #2

Lines 408/409: "In particular, in Scenario I, we observed a delay in the peak occurrence, i.e., there is a prolongation of the effects caused by the pandemic." This sentence is confusing and risky, because the reader would expect to see an estimation of the difference in the **incidence** peak, not the peak of the ratio predicted cases/observed cases, which might not coincide with peak incidence. Also, the peak for this of metric appears only in scenario 1, while the trend in scenario 2 is decreasing overall, therefore is not very straightforward to see where the peak is. I suggest the authors to either drop or clarify this message of peak delay.

Change #2

We thank the reviewer for raising this issue. To avoid misunderstanding, we followed the reviewer's suggestion and chose to remove this sentence from the current version of the manuscript.

Comment #3

Fig. 1: I am happy the authors now specify that the vertical lines represent the mean values, but it would be nice to read the value of both as well, since the xscales are different, therefore is not easy to evaluate them. I suggest the authors to provide the values of the means (possibly standard deviation as well) in the caption.

Change #3

Thank you for the suggestion. We revised the caption of the figure (Section **Methodology**, page 6), adding the mean and standard deviation measures of the distributions, as requested.

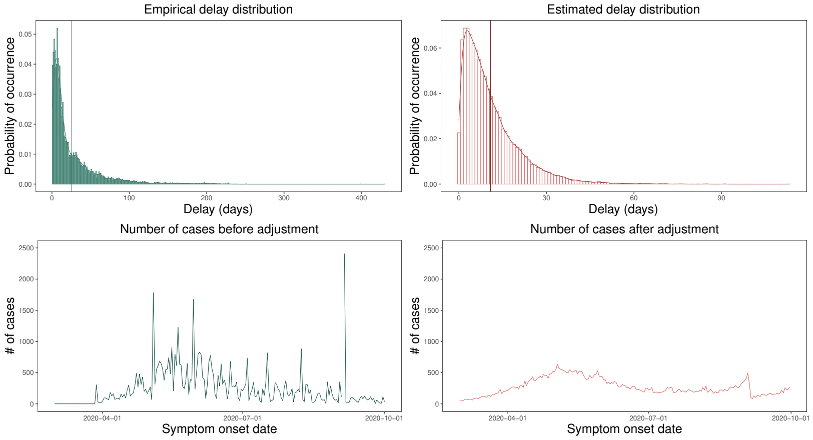

Fig. 2. **Probability distribution of the delay in the notified cases.** The distributions of delay and number of cases estimated from the data notified by Opendata SUS platform and the Coronavirus Panel before and after adjustments of the lag between symptom onset and official notification in Fortaleza/CE (Brazil). From right to left, we have: empirical delay distribution (with $\mu = 25.72$ and $\sigma = 31.85$)[2], estimated delay distribution (with $\mu = 10.85$ and $\sigma = 9.61$), cases distribution before fits, cases distribution after fits. The vertical lines on the first and second plots represent the mean of the delay in each analyzed distribution.

[2] μ is the mean of the distribution and σ is the standard deviation.

Comment #4

Fig. 4: Are those the posteriors for q1 and q2 in Fortaleza? Or is it a different city? Please specify.

Change #4

We appreciate the excellent observation. Although we have mentioned it in the text, such information was not specified in the figure. Therefore, we have modified the caption to state that the posteriors were estimated based on data from Fortaleza (Section **Case study I: Coarse-grained analysis**, page 12).

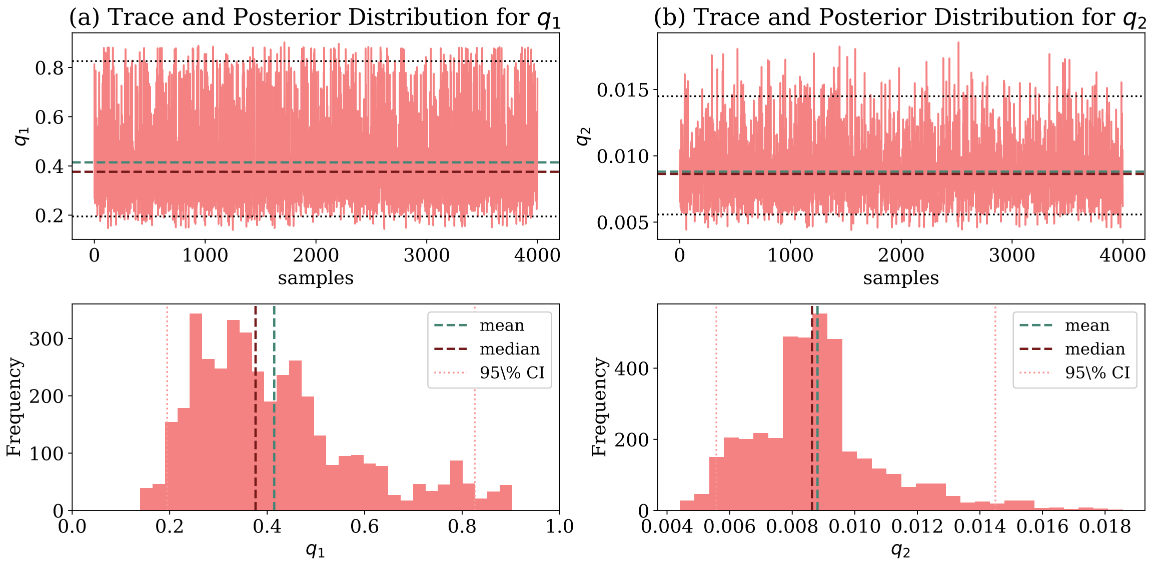

Fig. 3. **Mobility parameters estimated by our model for Fortaleza.** Estimated values of mobility parameters q_1 and q_2 via MCMC and their resulting distributions. (a) q_1 (From 03/15 to 03/20). (b) q_2 (From 03/20 to 05/05).

Comment #5

I think the caption of Fig.8 refers to Figure 9 and viceversa, please check.

Change #5

We carefully checked the captions and could not find any inconsistencies between them. So we believe they are correct.

Comment #6

Figure 9b: the line of DETRAN daily trajectories appears quite noisy, maybe the authors could to consider to add a moving averaged version for the sake of readability.

Change #6

We thank the reviewer for the suggestion. We added the moving average plot accordingly to the suggestion, as shown in figure below. In the paper, this figure is now named Figure 10.b.

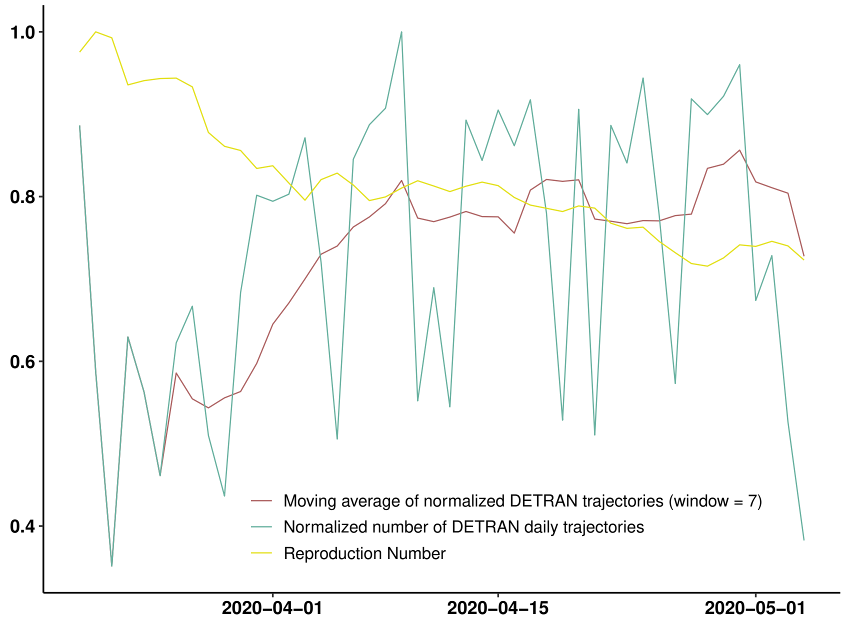

Fig. 4. Aggregate flow of vehicles, moving average aggregate flow of vehicles and the reproduction number between the dates 03/20/2020 and 05/04/2020.

Comment #1

The paper has improved after revision and the authors addressed all my comments, but I am not entirely convinced that the mobility matrix itself is so important here. I do understand that the idea is to put it so that it is possible to implement restriction scenarios, but this can be done with a time-dependent factor, such as q_t but not bounded by [0,1], as shown in the response letter. So, I wonder what happens to the results if, for example, the matrix elements are now random, keeping the total number of edges, weights, and nodes of the network; please see some network randomization procedures.

Change #1

Thank you for the comment and we agreement with the review. Initially, in the dataset adopted in this work, we observe that the number of DETRAN-CE daily trajectories decreases considerably on the day 2020-03-15 (beginnings of social isolation promotion), as can be seen in the figure below.

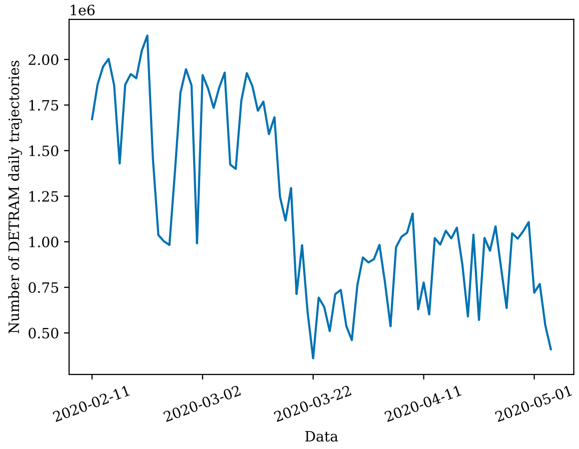

Fig. 5. **Number of DETRAN-CE daily trajectories.**

Thus, for this work, we assume as a premise that urban mobility before the announcement of the first cases of COVID was more intense than the period analyzed in the paper, referring to social isolation, thus justifying the choice of $0 \leq q_t \leq 1$. However, we can adopt a less restrictive range without losing the generalizability of the technique. Therefore, we include an assessment of our model using the DETRAN-CE dataset and $0 \leq q_t \leq 10$ from 03/15 to 03/20. Note that we have to provide a q_t prior distribution because we used a Bayesian estimation, the Stan simulation. Thus, we adopted $q_t \sim \mathcal{U}(0, 10)$, with $\mathcal{U}$ being the uniform distribution. Figure 6 shows the posterior distribution of q_1 when we adopted $0 \leq q_t \leq 10$. We observed that the estimated mean is 0.409, similar to 0.417, when we used $0 \leq q_t \leq 1$. This result suggests that increasing the interval of q_t has little influence on the outcomes for this dataset. This behavior corroborates our assumption that urban mobility was more intense before the pandemic began.

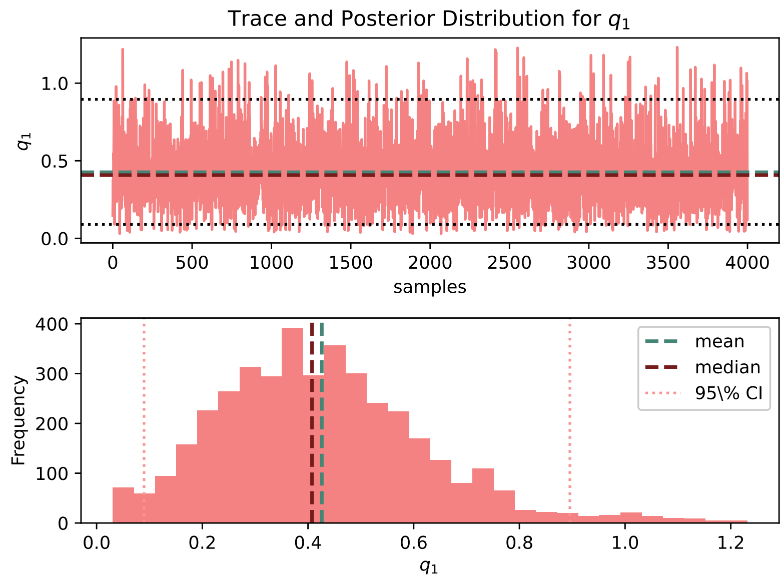

Fig. 6. **Mobility parameters estimated by our model for Fortaleza city.** Estimated values of mobility parameters q_1 (From 03/15 to 03/20) via MCMC and their resulting distributions.

In addition, we performed two new experiments for evaluation in scenarios in which the matrix C_{ij} was randomly shuffled and $0 \leq q_t \leq 1$:

- In the first scenario, we consider the extreme case in which we perform a random permutation of the matrix C_{ij}. More precisely, we perform a uniform random shuffle on each row of the matrix, so as to keep the row sums.
- In the second scenario, we use network randomization procedures based on the Markov Chain Approach, as seen in [1].

For the first scenario, we obtained the fit depicted below. This figure shows that the random matrix degrades the result, thus impairing the model's prediction.

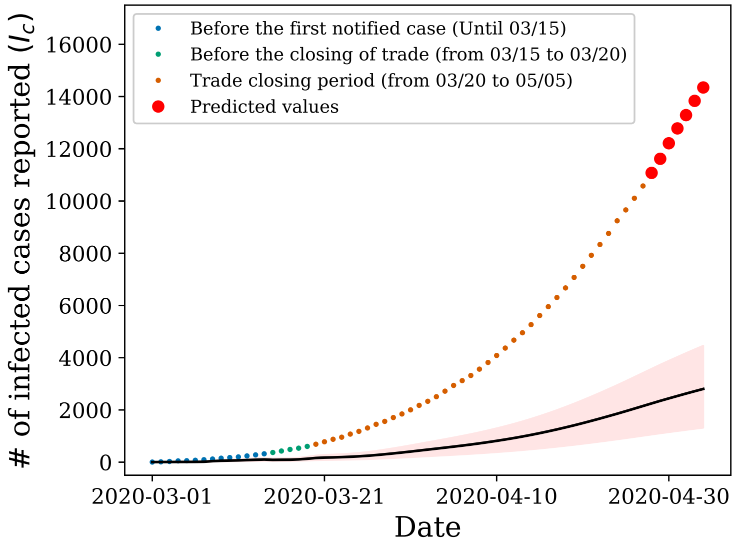

Fig. 7. **Model results for first scenario.** Our model applied to data from the city of Fortaleza. The shaded areas represent the 95 %confidence region provided by the model; the black line represents the average model prediction and the points the official values released.

We also observed (albeit attenuated) degradation in the result for the second scenario. Hence, even looking at the number of patients in the model's confidence interval, we see that the model's prediction significantly differs from the actual data. Finally, we showed our model applied to data from the city of Fortaleza to compared with the two scenarios, as can see below.

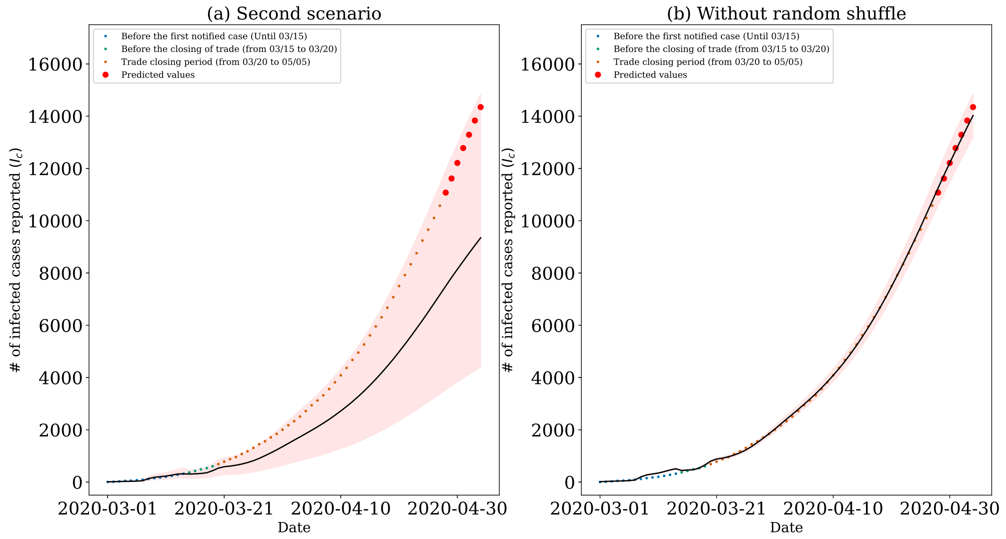

Fig. 8. **Model results.** Our model applied to data from the city of Fortaleza: (a) Second scenario and (b) C_{ij} without random shuffle. The shaded areas represent the 95 % confidence region provided by the model; the black line represents the average model prediction and the points the official values released.

We added in Section **SENUR model equipped with mobility information** on Page 7:

> *The impact of mobility on the virus spread at time t is given by the matrix* $W_{ij}(t) = q_t C_{ij}$*, where* $q_t \in [0, 1]$ *is a scalar, time-dependent parameter estimated by the model to quantify the influence of mobility on pandemic dynamics. Hence, we model the virus transmission by capturing people's mobility as they move from one neighborhood to another and exploring its relationship to the spread of the disease. Thus, for this work, we know a prior that mobility before the periods of isolation measures are more significant than the period analyzed in the article, thus justifying the choice of* $0 \leq q_t \leq 1$*. However, we can adopt a less restrictive range without losing the generalizability of the technique.*

Reference

[1] CARSTENS, Corrie Jacobien; BERGER, Annabell; STRONA, Giovanni. A unifying framework for fast randomization of ecological networks with fixed (node) degrees. **MethodsX**, v. 5, p. 773-780, 2018.

Comment #2

Also, I still find necessary some revisions on the presentation of the figures so that they have the same date format (some are YYYY-MM-DD and others MM/DD, some tick labels are rotated, others not).

Change #2

We corrected all data formats according to the suggestion, and we fixed all tick labels rotation.

Comment #3

By the way, there is a Ref. that can be interesting since the paper deals with data from Ceará: "Superspreading k-cores at the center of COVID-19 pandemic persistence", arXiv:2103.08685 (2021).

Change #3

Thank you for the suggestion. We added a citation to the aforementioned study in Section **Introduction** on Page 2:

> *Specifically focused on Brazil, several studies investigated the impact of human mobility on the spread of the disease using different types of data. For instance, some studies [14, 15] used Brazilian census information about people and terrestrial vehicles as well as air transportation data, basing the measurement of SARS-CoV-2 spreading patterns on data collected before the pandemic. Other studies calculated such spreading patterns between cities through mobile phone data [16-18]. Serafino et al. [19] implemented a protocol for optimized quarantines based on the analysis of contact tracking networks, in order to dismantle the coronavirus transmission chain with the minimum necessary interruptions. To monitor the evolution of the transmission contact network before and after quarantines, a compilation of hundreds of human mobility applications deployed in Latin America was used.*
